# Supplementary material for: Physiologic biomechanics enhance reproducible contractile development in a stem cell derived cardiac muscle platform
Source: Nat Commun. 2021 Oct 25;12:6167. doi: 10.1038/s41467-021-26496-1 (PMC8546060; doi:10.1038/s41467-021-26496-1)
Supplement: Supplementary file 1 — Supplementary Information [file 41467_2021_26496_MOESM1_ESM.pdf]

Supplementary Information

Supplemental Notes

Modeling Analysis of 2DMB Biomechanics on Elastic PDMS Substrates

A mechanics problem was set up to investigate the relationship between fractional shortening and force exerted by 2DMBs on elastomeric substrates composed of PDMS. In particular, we aimed to assess whether uncertainty in the mechanical characteristics of the 2DMB or variation in the dimensions of the PDMS influenced the relationship between fractional shortening and exerted force. The simulation was based on a single 2DMB adhered onto a PDMS substrate. To replicate the conditions of the experiments as closely as possible, the 2DMB was defined as a thin cuboid of 7:1 length to width aspect ratio - 308 µm length, 44 µm width, and 10 µm thickness. The buffering space between micropatterns was 120 µm in the long-axis direction and 80 µm in the short axis direction – corresponding to a PDMS:2DMB ratio of approximately 1.4:1 length ratio and 2.8:1 width ratio, leading to 431.2 µm length and 123.2 µm width for the PDMS. The PDMS thickness was set to 70 µm to match manual measurements of PDMS from fabricated devices.

The material properties of the PDMS were defined using a neo-Hookean constitutive model with stiffness 8 kPa.[1] Cardiomyocyte's stiffness can vary between 2 and 12 kPa, so here the 2DMB was modelled based on an underlying neo-Hookean model with stiffness 8 kPa. Fibers were prescribed along the length of the 2DMB. An active contraction maximum of 300 nN/µm^2 (mN/mm^2, or 300 kPa) was imposed along the fiber direction. This covers the range of typical myocardium contractility (~50 mN/mm^2). More complex passive cardiac mechanics models were feasible, but not considered here due to a lack of passive mechanical data and the amplification of the passive parameter space. Note that the aim was not to model the exact characteristics of the muscle strip. Instead, we wanted to capture the response of the gel substrate and relate it to force measurements generated by a generic muscle strip.

For modelling purposes, structural symmetry was assumed. Thus, only ¼ of the problem was simulated (½ of the length and ½ of the width), and the results were reflected in order to visualize the entire domain (Figure 2A). For the PDMS, a hexahedral mesh with 500 elements and 4851 quadrilateral nodes was created. For the 2DMB, a hexahedral mesh with 50 elements and 651 quadrilateral nodes was used. For the latter, some of the elements were modified into collapsed elements, as the short side of the 2DMB was modelled as tapering into the PDMS substrate, as can be seen in Figure 2A. On the bottom and sides of the PDMS, 0 displacement boundary conditions were imposed, whereas at the reflection planes, in both the PDMS and the 2DMB, no penetration was allowed. The displacements at the contact interface between the 2DMB and PDMS were restricted to be the same, i.e. the 2DMB could not slide on top of the PDMS. The force exerted by the 2DMB on the PDMS substrate was computed over the interface surface. The forces reported are computed as the integral of the traction forces over the interface surface between the 2DMB and PDMS (traction force). All simulations were run in CHeart.

Simulations were run to examine how the observed fractional shortening correlates with force, as well as to understand how varying the testing conditions might affect the observations. The effect of the PDMS buffering space on the fractional shortening was investigated by varying the PDMS:2DMB length ratio between 1.2:1 to 2:1 and the width ratio

was varied between 2:1 to 3:1, both with an increment of 0.2:1. Subsequently, the effect of PDMS thickness (varying between 20 and 100 µm) on fractional shortening was examined. Finally, the stiffness of the 2DMB was altered between 2 to 12 kPa, respectively, to study how the force vs fractional shortening behavior changes.

Although the mean fractional shortening observed in experiments was approximately 5%, the range extended to ~11%. Therefore, our simulations were performed up to a fractional shortening of ~11% to verify fidelity of the system across the entire range of experimental measurements. In Supplemental Figure 2, it can be seen that prescribing the PDMS buffer used in the experiments (1.4:1 and 2.8:1 PDMS:2DMB length and width ratios, respectively) leads to 11.2% fractional shortening (~17.2 µm total displacement, i.e. ~8.6 µm at each end) and 11.4 µN force exerted by the 2DMB. Increasing the buffer in either length or width would lead to a similar fractional shortening reading (11.4%, an increase of 0.2%), while decreasing the buffer space would also lead to similar values (e.g. 10.7%, a decrease of 0.5%). The force varies by less than 10% between the tightest and largest buffering spaces (12.1 to 11.1 µN). When the buffer space is small, the 2DMB contracts less and exerts more force on the PDMS, compared to a larger buffer space. However, the absolute reading differences would be even less significant when the typical maximum fractional shortenings observed are ~5%. Thus, it can be inferred that the buffer space used in the experiments does not alter the fractional shortening and forces, and adjacent 2DMBs are not likely to significantly influence each other.

The next test investigated the influence of PDMS thickness on relationship between force and fractional shortening. We calculated this relationship for 20, 30, 40, 70 and 100 µm PDMS thickness (Supplemental Figure 2). A thin PDMS layer, of only 20 or 30 µm, would lead to a biased relationship, whereas it can be seen that for PDMS thicknesses larger than 40 µm the behavior does not change significantly. Thus, the experimental substrate thickness (~70 µm) does not induce a bias on the behavior of force with fractional shortening, even if a fabrication error resulted in up to a 30 µM difference.

We selected 8 kPa as the elastic modulus of 2DMBs to model the above conditions, based on prior measurements in cardiomyocytes. As previously discussed, the fractional shortening of 2DMBs was computed based on displacement readings on top of each 2DMB. Since the actual elastic modulus may vary across individual 2DMBs, we tested how varying the elastic modulus might introduce error into calculations of force. As shown in Supplemental Figure 2, a lower 2DMB elastic modulus would result in a higher measured fractional shortening due to greater deformation of the muscle strip relative to the stiffer 8 kPa PDMS. In contrast, the behavior of 2DMBs with elastic moduli in the range of 8-12 kPa tends towards an asymptotic behavior. This analysis shows that variability in elastic moduli across individual 2DMBs in the range of 8-12 kPa will have a negligible effect on fractional shortening and force generation on 8 kPa PDMS, while tissue elastic moduli <6 kPa will result in systematic overestimation of traction forces.

Additionally, we analyzed how ROI placement for fractional shortening measurements may affect reliability of measurements. Because of edge tapering, the relative fractional shortening does not increase linearly near the 2DMB long-axis ends (Supplemental Figure 3D). Based on analysis of fractional shortening and force relationships, we selected placement of ROIs at the inner 50% of 2DMB length to minimize effects of ROI placement on measurements

of contractility, since fractional shortening measurements are relatively constant in these regions (Figure 2A, Supplemental Figure 3E-F).

Finally, we generated a MATLAB function to correlate fractional shortening and traction force based on the above results with inputs of PDMS stiffness and ROI location. Example correlations are shown in Table 1 summarizes the correspondence between fractional shortening and force for the case of 2DMBs on 70 µm thick, 8 kPa PDMS.

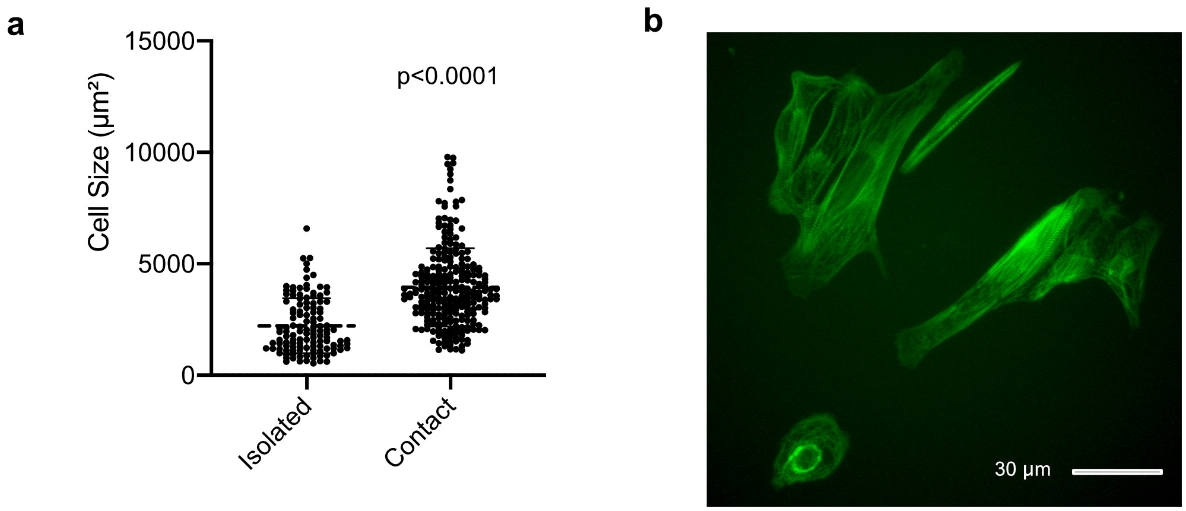

Supplemental Figure 1. Cell contact drives iPSC-CM growth. a Cell area of iPSC-CMs was measured from phalloidin-stained images of iPSC-CMs plated at intermediate density to control for effects of batch or media conditions. Cell area was analyzed to compare sizes of isolated single cells versus cells in contact with at least one other cell. **b** Representative image showing cell sizes in isolated iPSC-CMs compared to those in direct contact with other iPSC-CMs.

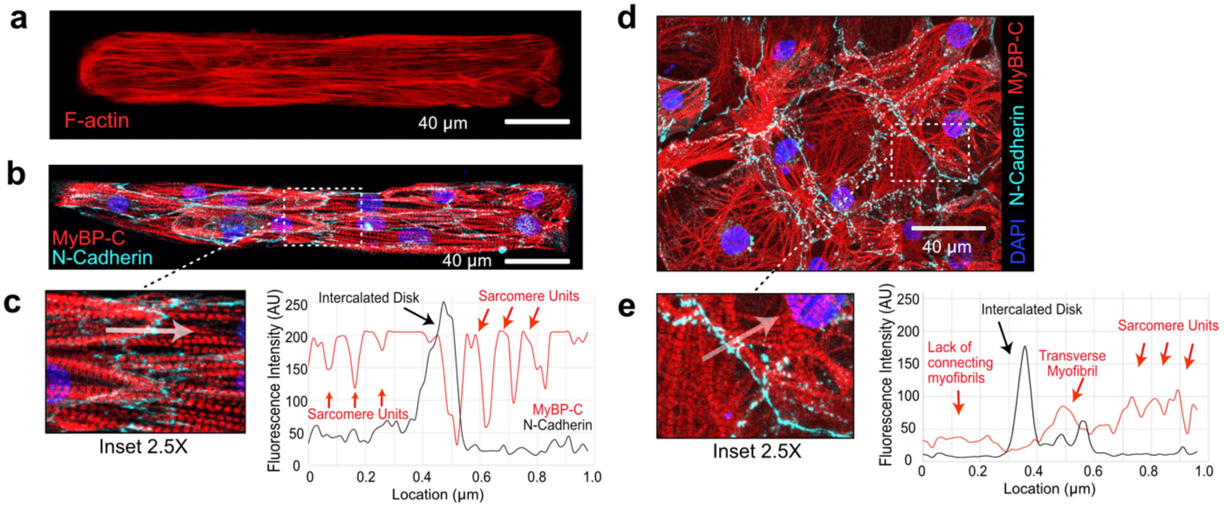

Supplemental Figure 2. Myofibrillar alignment rapidly develops in 2DMBs and is associated with organization of intercellular junctions. a Representative example of 2DMB showing myofibrillar alignment by 3 days following dissociation and re-plating (stained by SirActin to label F-actin). **b** Representative 2DMB showing myofibrillar organization at cell junctions by the intercalated disk protein, N-cadherin. **c** Inset (2.5X) and intensity profile (measured across opaque arrow) shows that myofibrillar bundles are aligned at cell junction connection points with continuation of sarcomeric periodicity across the border junction. **d** Representative standard iPSC-CMs, showing myofibrillar disorganization within and between cells that prevents consistent myofibrillar alignment across cell junctions. **e** Inset (2.5X) and intensity profile (measured across opaque arrow) shows that myofibrillar bundles are not consistently aligned at cell junction connection points in standard iPSC-CMs. In the region measured with the intensity profile, no aligned myofibrils are present left of the junction. Right of the junction, a myofibrillar bundle runs parallel to the junction, which is transverse to other myofibrillar bundles connecting to the junction (the perpendicular myofibrils exhibit sarcomeric periodicity along the profile).

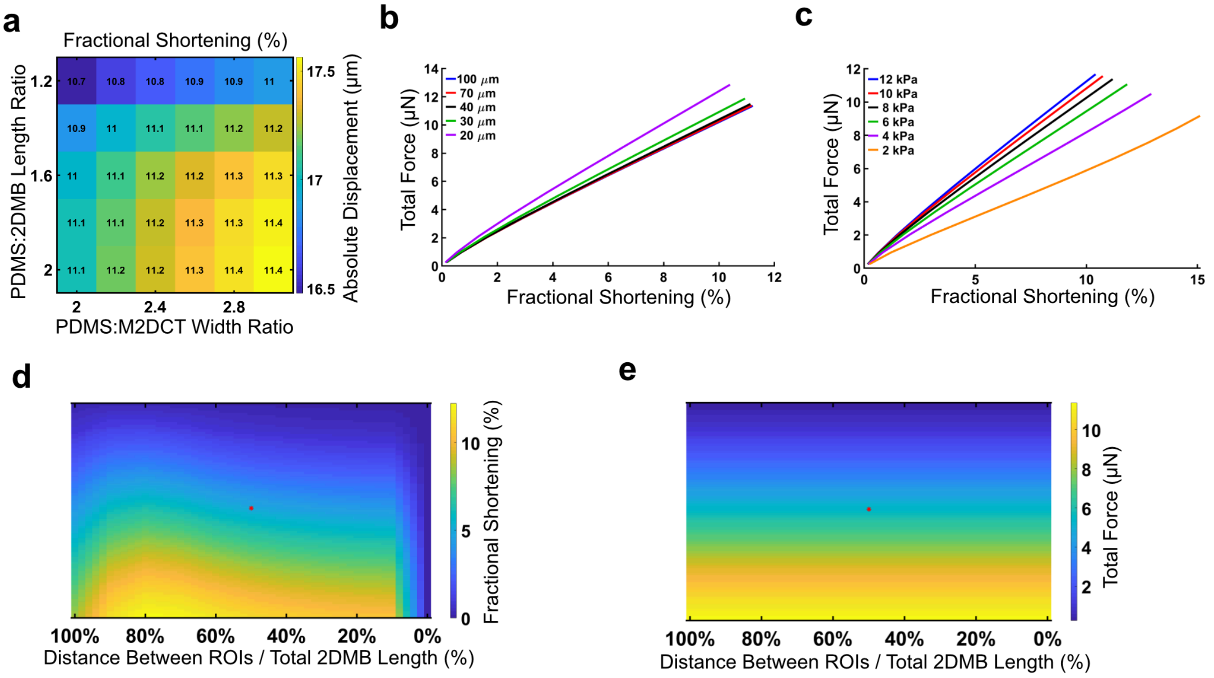

Supplemental Figure 3. Modeling shows minimal boundary effect and minimal impact of substrate thickness on 2DMB force development while enabling conversion of ROI displacements to total force. **a** The 2DMB substrate design includes a buffering region of PDMS surrounding each muscle bundle with a ratio of 1.4 of PDMS:2DMB along the bundle length and 2.8 along the bundle width. Modeling the deformation of 8 kPa PDMS by an 2DMB with 11% fractional shortening (>95th percentile of observed fractional shortening) demonstrates that further increasing the buffering region resulted in only minor changes in the relationship between force and bundle displacement. **b** Variation in PDMS thickness in the range of 40-100 µm demonstrates negligible effects on the fractional shortening and force relationship, while 30 µm thickness and lower requires greater force to achieve a given fractional shortening due to tethering from the underlying glass. **c** The effect of cardiomyocyte elastic modulus on underlying PDMS deformation and force generation at given fractional shortening magnitudes was assessed by modeling each using a neo-Hookean model. In the range of cardiac tissue elastic moduli (8-12 kPa), the influence of variability in 2DMBs' elastic moduli was minor on 8 kPa PDMS and relationships were approximately linear. **d, e** The effect of region of interest placement for tracking fractional shortening was evaluated by modeling the dispersion of regional displacements within an 2DMB to attain a given total traction force assuming homogeneous distribution of contracting myofibrils within the bundle and tapering of bundle edges at the lengthwise ends. d and e show corresponding fractional shortening (d) and total force (e) heat maps with local correlations as a function of distance between the measured ROIs on the x-axis. Near the lengthwise bundle edges (100%) and near the bundle center (0%), small differences in ROI placement exert a large influence on the correlation between fractional shortening and total force. We selected ROI placement to capture displacements of the inner 50% of 2DMBs since this location is robust to minor errors in ROI location (matching red asterisk in the center of each heat map corresponds to inner 50% ROI measurements of an 2DMB contracting with 5% fractional shortening to generate 5.48 µN of

total traction force on the PDMS surface. The relationships shown in d-e are incorporated into a MATLAB script with a tabulated look-up table that calculates total traction force as a function of PDMS stiffness, fractional shortening, and ROI location.

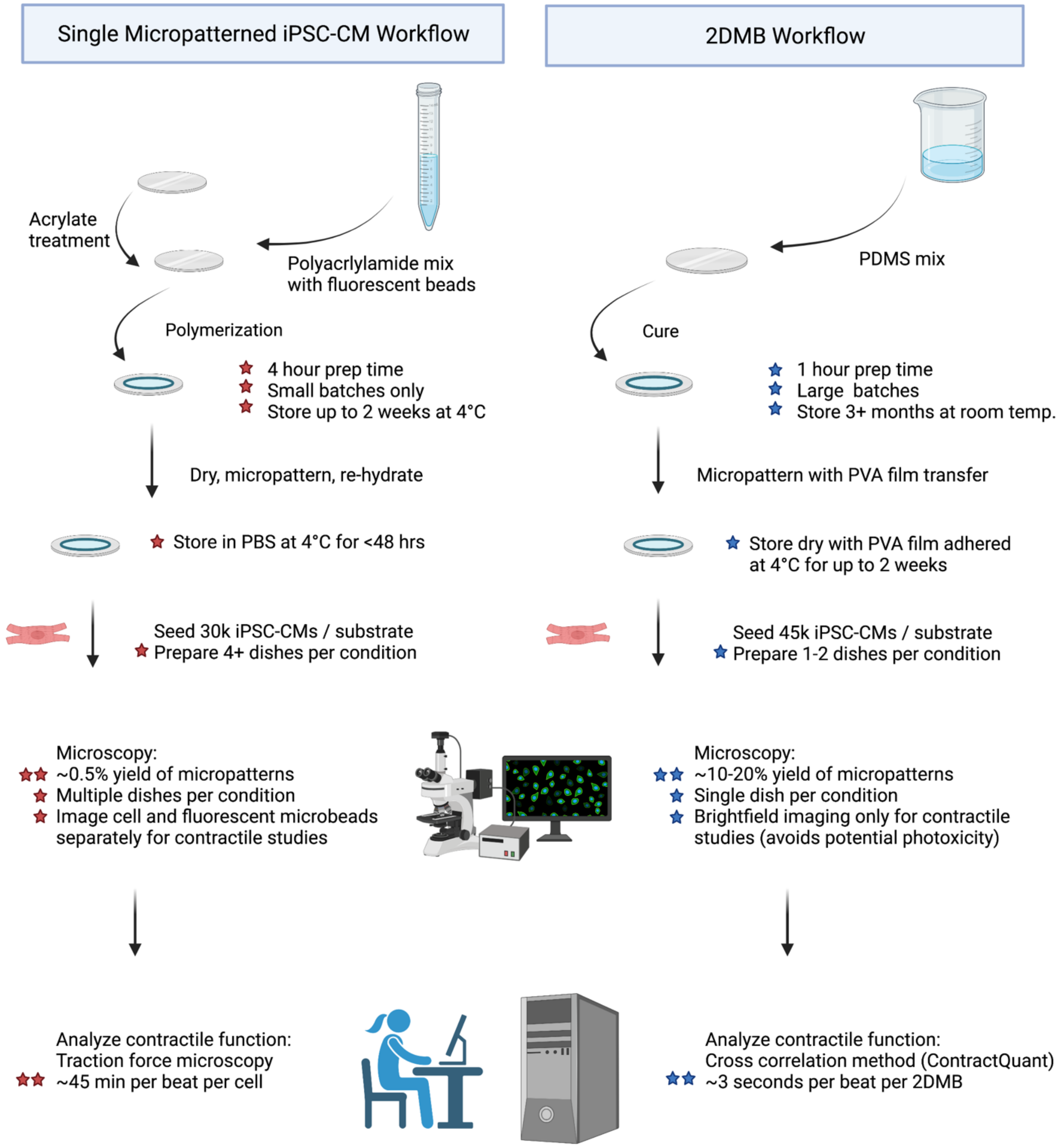

Supplemental Figure 4. Comparison of 2DMB and micropatterned single iPSC-CM workflow. Stars indicate steps in the protocol that have an improved workflow for 2DMBs, while 2 adjacent stars indicate major workflow improvements. Estimates of computational time were based on more than 100 separate analyses using an HP workstation with dual Intel Xeon E5-2630v4 2.2 2133 10 core processors (40 threads) and 64 GB RAM. Figure created with BioRender.

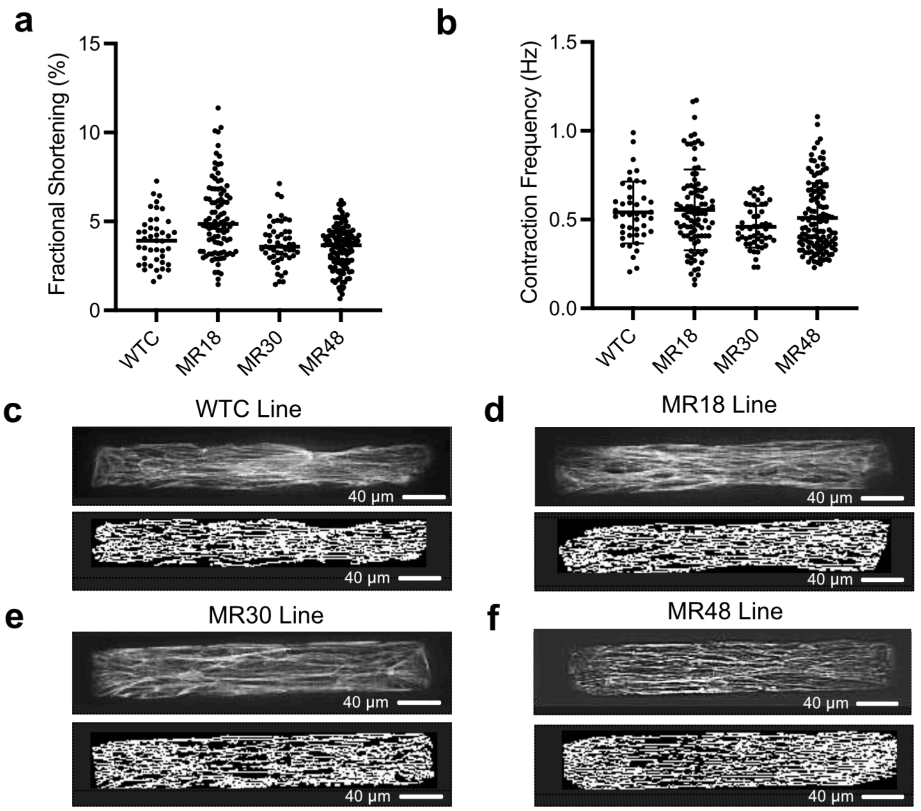

Supplemental Figure 5. Compatibility of 2DMB with multiple iPSC lines. a Additional iPSC lines were tested to demonstrate generalizability of the 2DMB platform. The WTC line and 3 randomly selected patient control lines all exhibited similar 2DMB formation on micropatterned substrates with uniaxial contractile direction and physiologic, auxotonic fractional shortening. **b** Spontaneous contraction frequency was generally similar among 2DMBs generated from each of 4 additional control lines. **c-f** Myofibrils aligned in the long axis direction for 2DMBs generated from the additional control lines and all were amenable to the 2D image processing algorithm, MyoQuant. The top image in each panel was obtained from the live cell F-actin dye (SiRactin) following contractile imaging, and the bottom image is the binary myofibrillar mask obtained from MyoQuant demonstrating long-axis myofibrillar alignment.

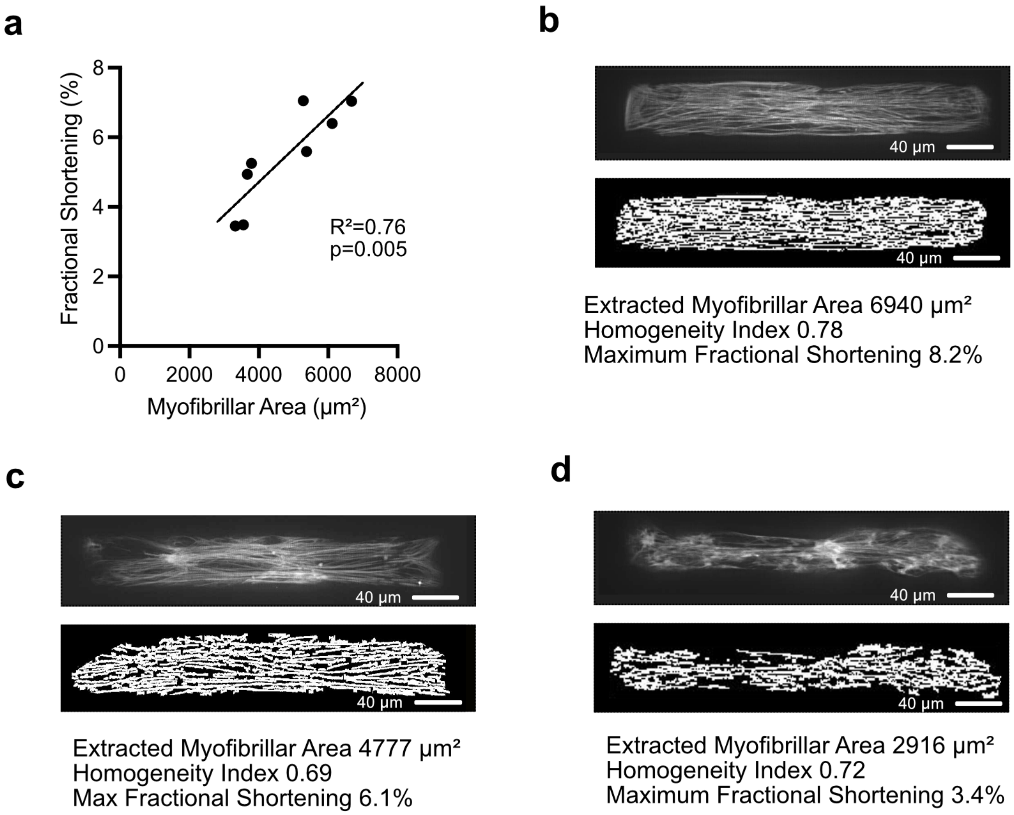

Supplemental Figure 6. Myofibrillar abundance correlates with differences in contractile function. A-C. Representative examples of 2DMBs with varying myofibrillar structural development. Myofibrils were imaged by labeling F-actin (SiR-actin) immediately following contractile imaging and then obtaining z-stacks of images. Automated analysis of deconvoluted images was performed in MATLAB to extract signal peaks from myofibrils, allowing calculation of total myofibrillar bundle area and an index of homogeneity of myofibrils within the 2DMBs. Greater myofibrillar area (as in A) generally correlated with larger magnitudes of fractional shortening. Poorly formed myofibrils (as in C) were present in a low proportion (<5%) of imaged 2DMBs, and these 2DMBs were excluded from analysis to avoid bias due to batch variability.

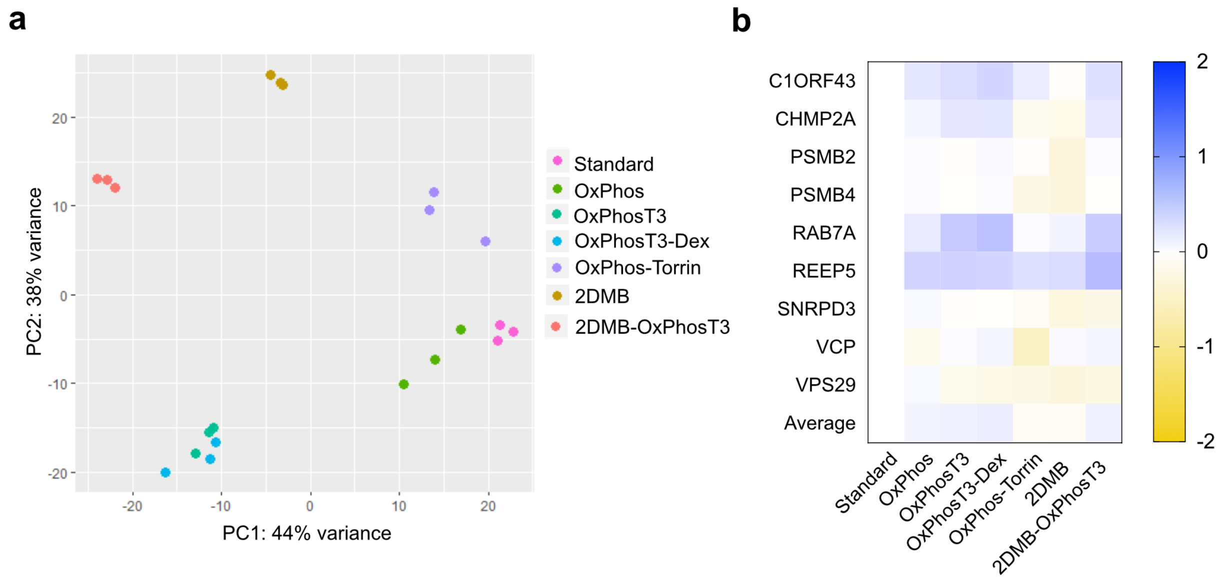

Supplemental Figure 7. RNA-Seq analysis reveals clustering between samples in different maturation conditions. a Principal component analysis of normalized RNA-seq reads across different maturation conditions shows independent clustering of biologic replicates (N=3) for each condition except for similar clustering among OxPhosT3 and OxPhosT3-Dex groups. **b** Relative expression (log-2 fold change) of a panel of housekeeping genes across the tested maturation conditions shows similar expression profiles indicating robust normalization of RNA-seq reads by DE-Seq2.

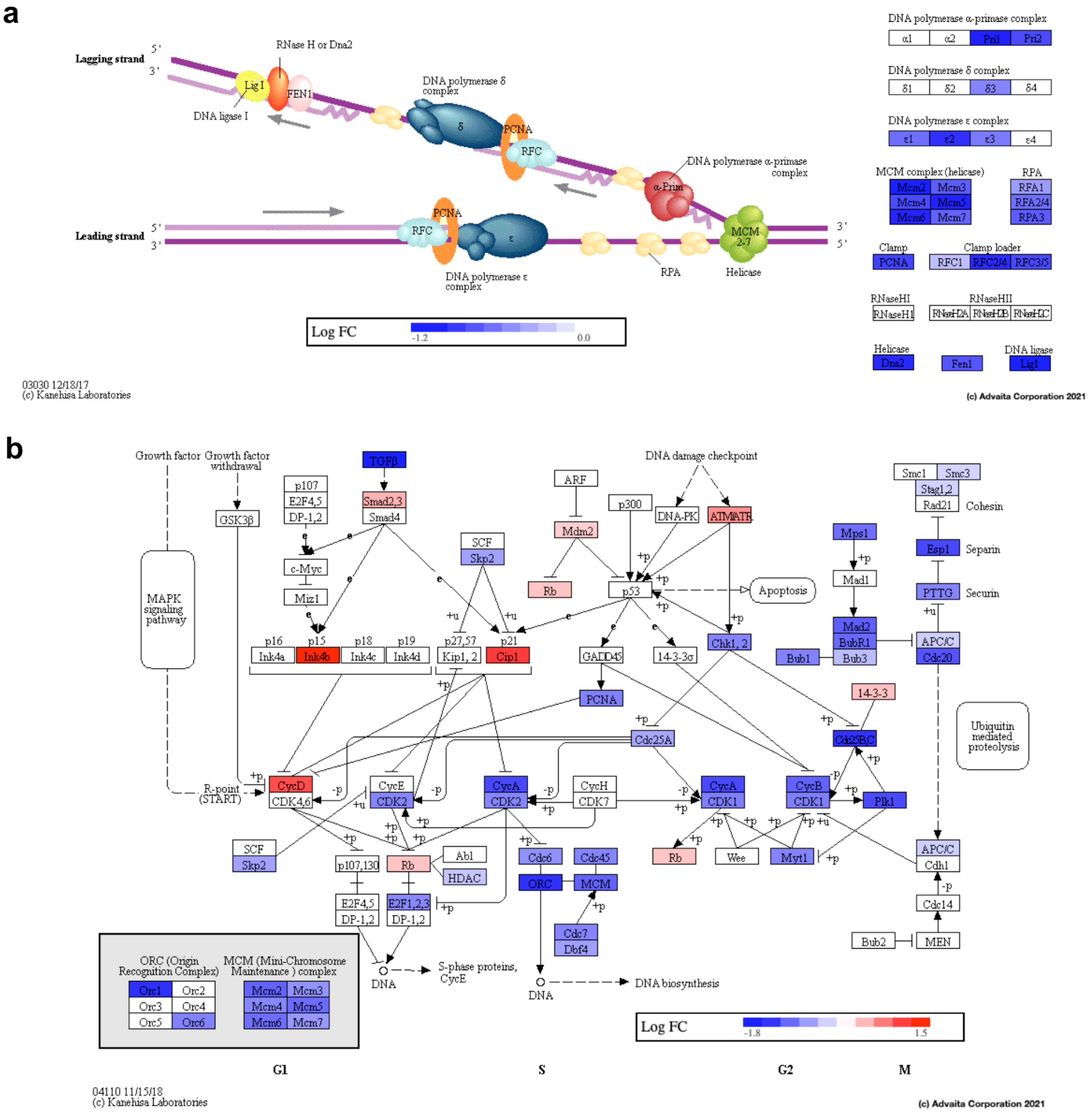

Supplemental Figure 8. RNA-Seq analysis reveals reduced DNA replication and cell proliferation gene expression in 2DMBs. a The Kegg pathway for DNA replication demonstrates down-regulation across most genes 2DMBs with log-fold changes relative to standard monolayers from the RNA-seq analysis (visualization from Advaita pathway analysis). **b** In addition to DNA replication genes, genes regulating cell proliferation were down-regulated in 2DMBs vs. standard monolayers, except for the negative regulators *CDKN1A* (encodes cip1) and *CDKN2B* (encodes ink4B). Visualization of Kegg pathway for RNA-seq data from Advaita pathway analysis.

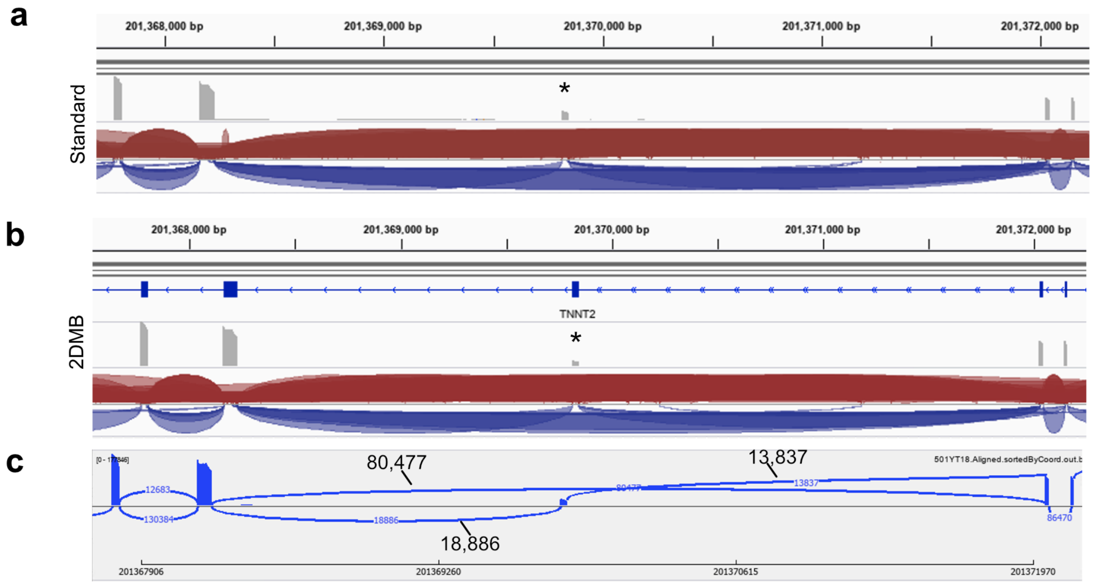

Supplemental Figure 9. 2DMBs exhibit a larger proportion of splice exclusion of exon 5 of TNNT2. a Read depth of RNA-seq reads for *TNNT2* surrounding exon 5 is shown for standard iPSC-CMs for a representative sample, shown from the IGV genome browser in the top track. In standard iPSC-CMs, 33% of TNNT2 transcripts contained exon 5 (PSI 32.6 ± 0.1%) indicating that 67% of transcripts are the adult variant (exon 5 marked by *). The lower track graphically depicts spice junction reads that include or exclude exon 5. **b** In 2DMBs, greater exclusion of exon 5 was present (PSI 17.4 ± 1.0%, p<0.0001) indicating that 83% of transcripts are the adult variant. **c** A Sashimi plot shows numbers of reads spanning splice junctions that either include or exclude exon 5 from a representative 2DMB.

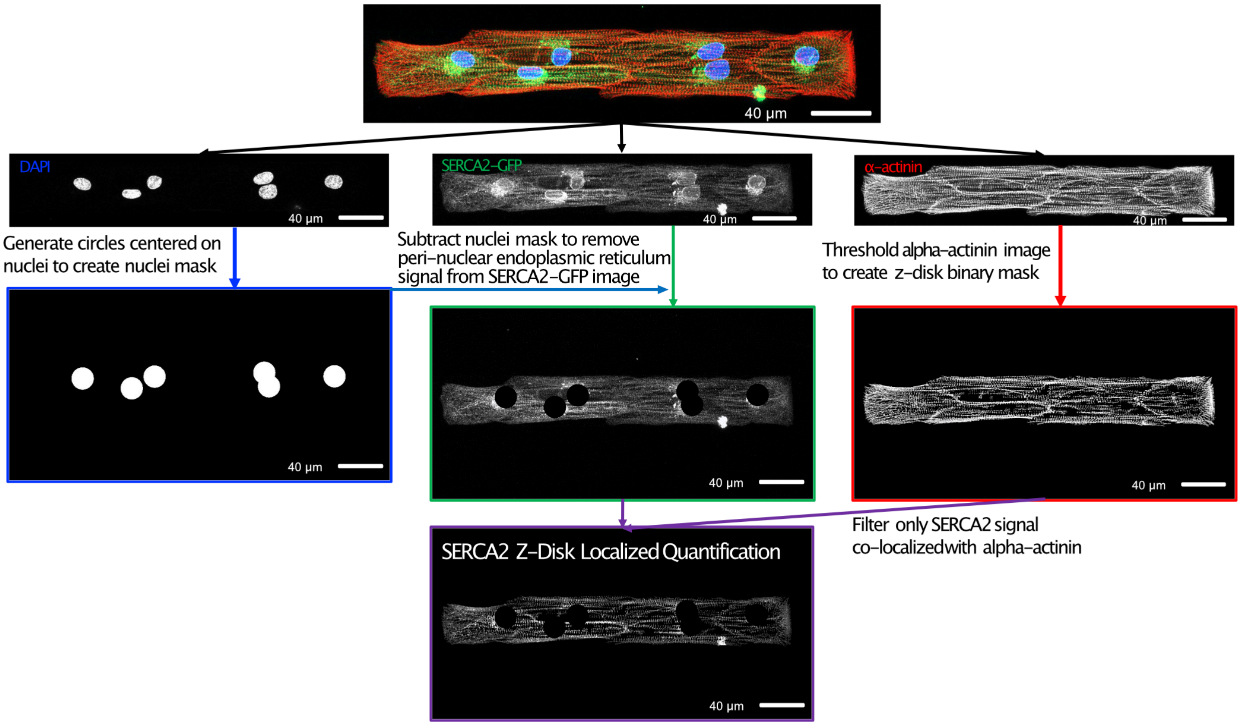

Supplemental Figure 10. SERCA2-GFP reporter line enables z-disk co-localization quantification in 2DMBs. Script were generated in Matlab to quantify z-disk localized SERCA2 from the SERCA2-GFP reporter iPSC-CMs in 2DMBs as shown in the algorithm overview. Since SERCA2 is processed in the endoplasmic reticulum, signal in the perinuclear region was removed by generating a nuclear subtraction mask from DAPI images. Alpha-actinin images were used to generate a binary mask representing locations of z-disks, where mature sarcoplasmic reticula structures are expected to localize. By filtering the SERCA2-GFP images for signal present specifically co-localized with alpha-actinin, z-disk localized SERCA2 signal was extracted for quantification across comparison groups. Images were obtained with a Zeiss linescan confocal microscope and maximum projection images were used for analysis.

a b

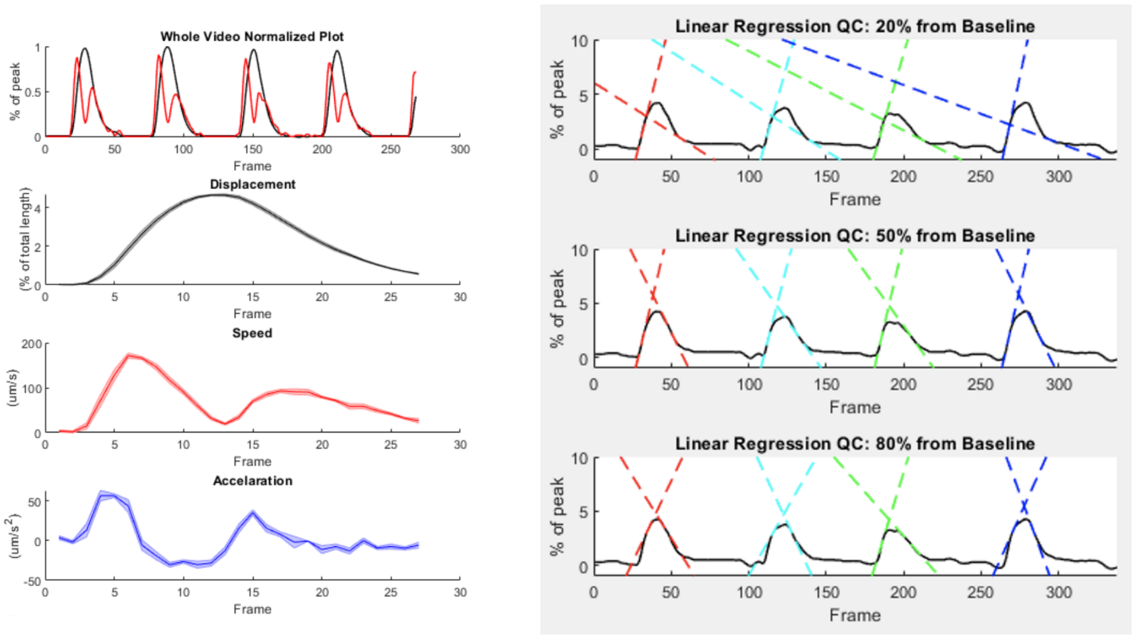

Supplemental Figure 11. ContractQuant enables kinetic analysis of *MYBPC3*$^{pr-/-}$ 2DMBs. a Example output from ContractQuant from an *MYBPC3*$^{pr-/-}$ 2DMB showing normalized displacement (black) and velocity (red, top row), fractional shortening (second row), velocity (third row), and acceleration (bottom row). The median value for each parameter is extracted for subsequent analyses. The merged contraction graphs (2nd-4th rows) generated by ContractQuant enable rapid verification of consistent motion tracking and symmetric muscle bundle contraction (solid lines indicate means and shaded regions 95% confidence intervals). **b** Example output from ContractQuant showing analysis of contraction and relaxation velocities from an *MYBPC3*$^{pr-/-}$ 2DMB at 20% (top), 50% (middle), and 80% (bottom) of peak contraction. The median value is extracted for subsequent analyses. The figures generated by ContractQuant enable rapid assessment of accurate automated identification of early, mid, and late time points for velocity calculations for each muscle bundle.
