# Supplementary material for: Physiologic biomechanics enhance reproducible contractile development in a stem cell derived cardiac muscle platform
Source: Nat Commun. 2021 Oct 25;12:6167. doi: 10.1038/s41467-021-26496-1 (PMC8546060; doi:10.1038/s41467-021-26496-1)
Supplement: Supplementary file 3 — Description of Additional Supplementary Files [file 41467_2021_26496_MOESM3_ESM.docx]

Description of Additional Supplementary Files

Title: Supplementary Data File 1.

Description: Gene Ontology – Biologic Process All Results

Title: Supplementary Data File 2.

Description: Gene Ontology – Biologic Process Summary Results

Title: Supplementary Video 1.

Description: Representative video of hPSC-CMs 48 hours after replating as 2DMBs to a micropatterned hydrogel. Although hPSC-CMs initially attach and assembly on micropatterns, delamination of the 2DMBs from the micropatterned surface occurs with progressive augmentation of contractile force during the first 48 hours.

Title: Supplementary Video 2.

Description: Representative large field of view (10X) of 2DMBs on micropatterned PDMS. Nearly all micropatterns exhibit attachment of at least 3 hPSC-CMs that form functional muscle bundles with uniaxial contractile direction, while most exhibit attachment of the goal 6-12 hPSC-CMs per micropattern, creating functionally independent 2DMBs, each with its own spontaneous contraction frequency. Contractions of each 2DMB are observed to not physically deflect or interact with adjacent 2DMBs, consistent with modeling analyses. Annotated black rectangles indicate 2DMBs with 6-12 cells, uniform geometries, and no overlying debris that would be suitable to contractile imaging at higher magnification.

Title: Supplementary Video 3.

Description: Representative video of an 2DMB from brightfield imaging at 40X. 2DMBs typically exhibit spontaneous contractions with symmetric and homogeneous contractions in the uniaxial direction of the 2DMB long axis.

Title: Supplementary Video 4.

Description: Representative video of an 2DMB imaged at 40X with an F-actin live-cell permeable stain (SiR-Actin) to visualize long-axis orientation of myofibrils during contractions. Desmoplakin-GFP reporter hPSC-CMs were used for this experiment, enabling concomitant imaging of intercalated disks (see Supplemental Video 5).

Title: Supplementary Video 5.

Description: Representative video of the same 2DMB as in Supplemental Video 4, showing that intercalated disks are located at cell-cell junctions at myofibrillar connection points. Intercalated disks are visualized by desmoplakin-GFP reporter hPSC-CMs (see also Supplemental Video 5 and Supplemental Figure 2).

Title: Supplementary Video 6.

Description: Live imaging of myofibrils from standard (nonpatterned) iPSC-CMs. Myofibrils were marked for F-actin (SiR-actin) for live-cell imaging. Regional heterogeneity in magnitude of contractions causes some regions to experience myofibrillar stretch due electromechanical coupling to adjacent, stronger-contracting cells.

Title: Supplementary Video 7.

Description: Representative large field of view (10X) of single hPSC-CMs on micropatterned PDMS using otherwise same methods as for 2DMBs on PDMS. The yield of single hPSC-CMs that uniformly fill single cell sized rectangles and exhibit spontaneous uniaxial contractions is typically low, requiring multiple substrates to be analyzed per experimental condition. Although hPSC-CMs were filtered prior to seeding, many micropatterns are adhered with >1 hPSC-CM while many others have no attached cells. Among attached cells, greater variability in hPSC-CM myofibrillar growth is observed (see also Figure 2G). Annotated black rectangles single micropatterned hPSC-CMs with uniform geometries that would be suitable to contractile imaging at higher magnification. Field of view size is the same as in Supplementary Video 2.
