# Supplementary material for: Physiologic biomechanics enhance reproducible contractile development in a stem cell derived cardiac muscle platform
Source: Nat Commun. 2021 Oct 25;12:6167. doi: 10.1038/s41467-021-26496-1 (PMC8546060; doi:10.1038/s41467-021-26496-1)
Supplement: Supplementary file 13 — Reporting Summary [file 41467_2021_26496_MOESM13_ESM.pdf]

nature portfolio

Corresponding author(s): Adam Helms
Last updated by author(s): Sep 20, 2021

Reporting Summary

Nature Portfolio wishes to improve the reproducibility of the work that we publish. This form provides structure for consistency and transparency in reporting. For further information on Nature Portfolio policies, see our Editorial Policies and the Editorial Policy Checklist.

Statistics

For all statistical analyses, confirm that the following items are present in the figure legend, table legend, main text, or Methods section.

n/a	Confirmed	
☐	☒	The exact sample size (*n*) for each experimental group/condition, given as a discrete number and unit of measurement
☐	☒	A statement on whether measurements were taken from distinct samples or whether the same sample was measured repeatedly
☐	☒	The statistical test(s) used AND whether they are one- or two-sided<br>*Only common tests should be described solely by name; describe more complex techniques in the Methods section.*
☐	☒	A description of all covariates tested
☐	☒	A description of any assumptions or corrections, such as tests of normality and adjustment for multiple comparisons
☐	☒	A full description of the statistical parameters including central tendency (e.g. means) or other basic estimates (e.g. regression coefficient) AND variation (e.g. standard deviation) or associated estimates of uncertainty (e.g. confidence intervals)
☐	☒	For null hypothesis testing, the test statistic (e.g. *F*, *t*, *r*) with confidence intervals, effect sizes, degrees of freedom and *P* value noted<br>*Give P values as exact values whenever suitable.*
☒	☐	For Bayesian analysis, information on the choice of priors and Markov chain Monte Carlo settings
☐	☒	For hierarchical and complex designs, identification of the appropriate level for tests and full reporting of outcomes
☒	☐	Estimates of effect sizes (e.g. Cohen's *d*, Pearson's *r*), indicating how they were calculated

Our web collection on statistics for biologists contains articles on many of the points above.

Software and code

Policy information about availability of computer code

Data collection	Microscopy images were obtained using NIS-Elements (Ver5.02.03). Data was recorded and organized in Microsoft Excel (ver 16.51).
Data analysis	Microscopy images were prepared for publication using Image J (ver 1.8). Images were quantified using custom scripts implemented in Matlab (ver R2018A). Specifically, contractile function was analyzed using the ContractQuant algorithm (https://github.com/Cardiomyocyte-Imaging-Analysis/ContractQuant.git) and myofibrillar quantification was performed with MyoQuant (https://github.com/Cardiomyocyte-Imaging-Analysis/MyofiberQuant.git). Sarcomere orientation (as in Figure 1) was performed with separate custom Matlab scripts as described by DePalma, et al. (PMID 33325920). Patch clamping data was analyzed with pClamp (ver 10). RNA-seq reads were aligned using STAR (ver 2.7.2, https://github.com/alexdobin/STAR). Normalization and quantification of RNA-seq data was performed using DE-Seq2 (https://github.com/mikelove/DESeq2). Other than RNA-seq, statistical analyses were performed in GraphPad Prism (ver 9).

For manuscripts utilizing custom algorithms or software that are central to the research but not yet described in published literature, software must be made available to editors and reviewers. We strongly encourage code deposition in a community repository (e.g. GitHub). See the Nature Portfolio guidelines for submitting code & software for further information.

Data

Policy information about availability of data

All manuscripts must include a data availability statement. This statement should provide the following information, where applicable:

- Accession codes, unique identifiers, or web links for publicly available datasets
- A description of any restrictions on data availability
- For clinical datasets or third party data, please ensure that the statement adheres to our policy

The RNA-seq data in this publication have been deposited in the GEO database under accession code GSE183398. Other datasets added in the Source Data file.

Field-specific reporting

Please select the one below that is the best fit for your research. If you are not sure, read the appropriate sections before making your selection.

☒ Life sciences ☐ Behavioural & social sciences ☐ Ecological, evolutionary & environmental sciences

For a reference copy of the document with all sections, see nature.com/documents/nr-reporting-summary-flat.pdf

Life sciences study design

All studies must disclose on these points even when the disclosure is negative.

Sample size	Sample sizes for each experiment were chosen empirically to include at least 10 separate biologic replicates for individual 2DMBs. Based on post-hoc analysis, the standard deviations for most measurements using this number of replicates yielded statistical power >0.9 with alpha <0.05. For pooled 2DMBs (e.g. RNA-seq data), each biologic replicate consisted of thousands of 2DMBs pooled from multiple differentiation batches, and 3 such samples were used as the biologic replicates for analysis. Confounding variability was reduced for the RNA-seq experiment since experimental conditions were imposed on replicates obtained from the same batches and treated concurrently.
Data exclusions	2DMBs were not quantified unless they met the quality inclusion criteria described in the methods and shown in the Supplemental videos, including requirements for cell numbers per 2DMB and adequately filling the micropatterned regions. No data on 2DMBs meeting these metrics were excluded.
Replication	All data shown were replicated in at least 3 experiments except where stated in figure legends. The RNA-seq data was obtained from a single large experiment due to the cost of the method - in this case, biologic samples were pooled from multiple batches of iPSC-CMs to ensure that samples were representative.
Randomization	For all comparisons, iPSC-CM samples were randomly allocated into experimental groups from the same batches of cells to prevent batch variation influencing results. For pharmacologic studies (Figure 3), paired analyses were used such that drug responses were quantified in the same 2DMBs.
Blinding	Blinded and automated quantification was used for most analyses throughout the study to avoid bias. This included automated batch quantification of contractile function and cardiomyocyte structures.

Reporting for specific materials, systems and methods

We require information from authors about some types of materials, experimental systems and methods used in many studies. Here, indicate whether each material, system or method listed is relevant to your study. If you are not sure if a list item applies to your research, read the appropriate section before selecting a response.

Materials & experimental systems

n/a	Involved in the study	
☐	☒	Antibodies
☐	☒	Eukaryotic cell lines
☒	☐	Palaeontology and archaeology
☒	☐	Animals and other organisms
☒	☐	Human research participants
☒	☐	Clinical data
☒	☐	Dual use research of concern

Methods

n/a	Involved in the study	
☒	☐	ChIP-seq
☒	☐	Flow cytometry
☒	☐	MRI-based neuroimaging

Antibodies

Antibodies used	N-cadherin antibody (mouse 1:200, BD Biosciences AB_398236, clone 32); custom MyBP-C antibody (N-terminal rabbit polyclonal, 1:1000, generated in the lab of Samantha Harris); alpha-actinin antibody (mouse 1:1000, Sigma A7811, clone EA-53); Mlc-2v antibody (Proteintech rabbit polyclonal 10906-1-AP, 1:500). Secondary antibodies were goat anti-mouse AlexaFluor 488 (ThermoFisher 1:1000) and goat anti-rabbit AlexaFluor 568 (ThermoFisher 1:1000).
Validation	All antibodies were used for immunofluorescent imaging with Alexa Fluor secondaries. Antibodies were applied to human iPSC-derived cardiomyocytes. All of the antibodies used yield high signal:noise specific to the expected location of the corresponding sarcomere or intercalated disk structures and negligible background signal outside of these structures.

Eukaryotic cell lines

Policy information about cell lines

Cell line source(s)	Induced pluripotent stem cell lines: DF19-9-11 line from WiCell; GM25256 (WTC-11) line from Coriell; MR18, MR30, MR48

	lines from NYSCF Research Institute Stem Cell Repository; connexin-43-GFP/AICS-0053 cl.16 line, SERCA2-GFP/AICS-0046 cl.51 line, DSP-GFP/AICS-0017 cl.65 line from the Allen Institute.
Authentication	Lines obtained from repositories were authenticated by their respective repository for normal karyotype and pluripotency. Lines were maintained within 15 passages of initial vial obtained from the repository to minimize the likelihood of genomic alterations during prolonged passaging.
Mycoplasma contamination	All cell lines tested negative for mycoplasma contamination.
Commonly misidentified lines (See ICLAC register)	No commonly misidentified cell lines were used in the study.
